# Primary Care Comprehensiveness Can Reduce Emergency Department Visits and Hospitalization in People with Hypertension in South Korea

**DOI:** 10.3390/ijerph15020272

**Published:** 2018-02-05

**Authors:** Nak-Jin Sung, Yong-Jun Choi, Jae-Ho Lee

**Affiliations:** 1 Department of Family Medicine, Dongguk University Ilsan Hospital, Goyang 10326, Korea; snj@dongguk.ac.kr; 2 Department of Social and Preventive Medicine and Health Services Research Center, College of Medicine, Hallym University, Chuncheon 24252, Korea; ychoi@hallym.ac.kr; 3 Department of Family Medicine, The Catholic University of Korea College of Medicine, Seoul 06591, Korea

**Keywords:** primary health care, chronic disease, hypertension, usual source of care, Korea

## Abstract

Hypertension has been the leading risk factor contributing to cardiovascular morbidity and mortality, which needs comprehensive measures to manage and can be controlled effectively in primary care. In the health care context of South Korea, where specialists can see patients directly at their own community clinics and there has been no consensus on the definition of primary care, the authors used the nationally representative 2013 Korea Health Panel data, categorized adults (≥18 years) with hypertension by types of usual source of care (USC), and analyzed the association of having a comprehensive community clinic (i.e., primary care) physician as a USC with experience of emergency department (ED) visits and hospitalization within a year. After adjusting for cofounding variables including Charlson comorbidity index scores, those having a primary care physician as a USC remained associated with a decrease in an experience of ED visits (OR: 0.61, 95% CI: 0.40–0.93) and hospitalization (OR: 0.69, 95% CI: 0.49–0.96), compared to those not having a usual physician. Health policies that promote having a primary care physician as a USC could decrease unnecessary experience of ED visits and hospitalization by adults with hypertension. This can partly reduce ED overcrowding and avoidable hospitalization in Korea.

## 1. Introduction

Chronic diseases are the leading causes of death and disability worldwide. Disease rates from these conditions are accelerating globally, advancing across every region and pervading all socioeconomic classes. Cardiovascular disease, cancers, chronic respiratory diseases, and diabetes are categorized as the four main types of chronic disease. Hypertension has been proved as the major risk factor of cardiovascular morbidity and mortality [1]. Hypertension occurs in 69% of those with a first myocardial infarction, in 77% of those with a first stroke, 74% of those with chronic heart failure, and in 60% of those with peripheral arterial diseases [2]. It is one of the most prevalent chronic diseases also in Korea—among those aged 30 and older, 32.7% in men and 23.1% in women have hypertension [3]. There is still much room for improvement in managing patients with hypertension. Among adults with hypertension aged 18 and older in the United States of America (USA), prevalence of controlled hypertension during 2011–2014 was 53.0% [4]. Among Korean adults with hypertension, about 66% were aware of their diseases, about 64% took anti-hypertensive medications, and only 46% had controlled hypertension [3].

Hypertension is a disease that needs to be managed by comprehensive measures [5]. In this respect, the majority of cases of hypertension can be managed effectively at the primary care level. Primary care physicians (PCPs) as well as trained non-physician health workers can play a very critical role in detection and management of hypertension. Large multispecialty group practices in which PCPs play a central role have achieved high-quality and low-cost care for patients with chronic diseases [6]. One of the hallmarks of primary care is a role as a usual source of care (USC), which is the particular medical professional, doctor’s office, clinic, health center, or other place where a person would usually visit if sick or in need of advice about his or her health [7]. Individuals with a USC have had an association with increased accessibility to healthcare or health education, more use of preventive services, improved health status, and increased satisfaction with healthcare. Having a USC for hypertensive patients was associated with a higher percentage use of anti-hypertensive medication and better control of blood pressure [6,7,8,9]. Identification of a particular practitioner of care (a usual physician) has particularly been associated with better services, such as better recognition of problems or needs, more accurate or earlier diagnoses, a lower rate of emergency department use, fewer hospitalizations, lower costs, better monitoring, fewer drug prescriptions, fewer unmet needs, and increased satisfaction than mere identification of a particular place of care (a usual place only) [10,11,12,13].

Among the Organization for Economic Co-operation and Development (OECD) countries, Korea has one of the weakest health systems in terms of primary care orientation [14], although the national health insurance system has been operated since 1989. Medical specialists who operate their own community clinics (mostly solo practices, 93.5% in 2008 [15]) see patients directly, without referrals from family physicians, as there is no gatekeeping function for primary care in Korea. A lack of gatekeeping leads to competition among physician clinics and hospitals in the background of private sector dominance (>90% of all facilities) in the health care system. The method of remuneration is mainly based on fee-for-service so that physicians lack an incentive to focus on reducing unnecessary services and focusing more on disease prevention and health promotion [16,17]. A national representative survey in 2012 reported that only 13.9% of Korean adults aged 18 years and older had a physician as a USC [18], in contrast with the proportions of other countries: the USA 80%, Canada 84%, Australia 88%, the United Kingdom and New Zealand 89% each, Germany 92%, and the Netherlands 100% [19]. It seems natural that Korea had the highest frequency (16.0) of doctor consultations per person among the OECD countries compared with the OECD average of 6.9 per person per year in 2015 [20].

The Korean government attempted to introduce a primary care enrollment scheme in 1996, but this effort failed due to the strong opposition of the Korean Medical Association, absence in public opinion formation, insufficient government commitment, and scant scientific evidence on the benefits of primary care in South Korea. Since then, there has been no significant change in Korean primary care. However, considering a rapidly aging population and the increased prevalence of chronic diseases, the new administration led by President Moon Jae-in is willing to reform health care system toward having a primary care orientation. As primary care policy researchers, the authors planned to provide evidence of the value of primary care in health to guide political decision. We limited study subjects to people with hypertension. Hypertensive patients are adequate subjects for evaluating the effects of USC on the outcome, especially from the aspect of comprehensiveness because they need comprehensive measures to treat [5]. Comprehensiveness is a major attribute of primary care that differentiates a potential primary care community clinic from a non-primary care specialist community clinic in Korea [21,22].

## 2. Materials and Methods

### 2.1. Data Source

The authors used data from the Korea Health Panel (KHP) survey that is conducted annually by a consortium of the Korea Institute for Health and Social Affairs and National Health Insurance Corporation. In order to select a nationally representative sample of households, a multistage stratified probability sampling design is applied in the KHP survey [23]. The initial 2008 baseline data included 21,283 individuals in 7009 households, with 14,839 individuals in 5200 households remaining in 2013.

### 2.2. Sample Selection Process

**Figure 1:**
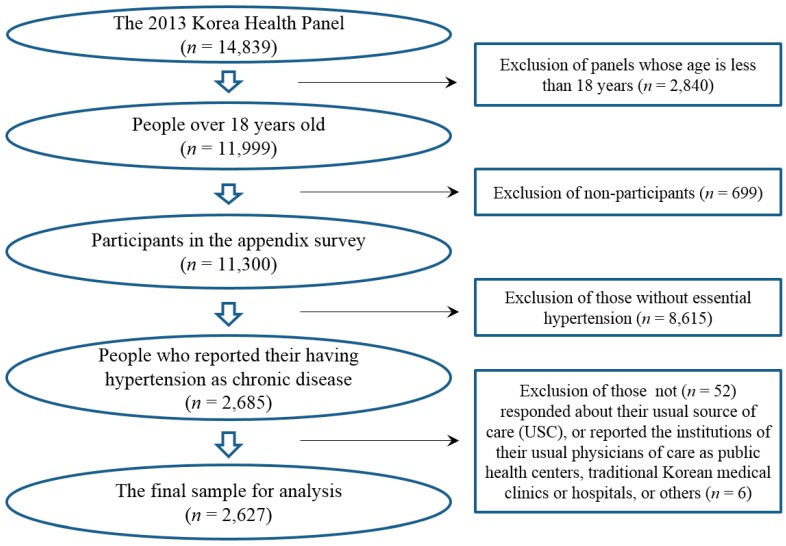
Sample selection process in this study.

Among total participants (*n* = 14,839) in the 2013 KHP survey, adults aged 18 years or older (*n* = 11,999) were eligible to participate in the appendix survey containing items about USC. Actual participants in the appendix survey were 11,300 (non-participation rate = 5.8%). Among these participants, 2685 reported that they had essential hypertension as a chronic disease (I10 by the 10th revision of the International Classification of Disease (ICD)). Fifty-two panels were excluded because they did not respond about having or not their usual place of care. We excluded an additional 6 panels who reported the institutions of their usual physicians of care as public health centers, traditional Korean medical clinics or hospitals, or others. Eventually, 2627 survey respondents were included for analysis in this cross-sectional study (Figure 1).

### 2.3. Measurement

#### 2.3.1. Sociodemographic Variables

Age was categorized into 3 groups: 18–49, 50–64, and 65 years or more; marital status into 2 groups: married and others (married and others—divorced, separated, widowed, or never married); education period into 3 groups: elementary school (0–6 years), middle or high school (7–12 years), and college or over (13 years or more); types of health insurance into 3 groups: employed, self-employed, and Medical Aid or others; and household income into 5 quintile groups.

#### 2.3.2. Self-Rated Health Variable

Self-rated health (SRH) was divided into 5 levels on a Likert scale (excellent, good, moderate, poor, and very poor) at the KHP survey, but it was combined into 3 levels (good, moderate, and poor) to interpret study results easily.

#### 2.3.3. Charlson Comorbidity Index

The authors used Charlson comorbidity index (CCI) to adjust for health status objectively in the sampled adults with hypertension. CCI is one of the measures for health status to categorize comorbidities of patients based on ICD diagnosis [24]. Each comorbidity category is assigned an associated weight (from 1 to 6), depending on the risk of dying associated with each one. Scores are summed to provide a single comorbidity score to predict mortality. The higher the score, the more likely the predicted outcome would result in mortality or higher resource use. The original index was developed with 19 categories. However, it has since been modified to 17 categories [25]. The list of specific diagnosis codes has been updated to be consistent with ICD-10 coding [26]. In this study, the authors could not include 2 categories (end organ damages of diabetes and moderate or severe liver diseases) in the calculation of CCI Score, because the KHP data did not provide diagnostic codes for these categories. Thyroid cancer (C73) had an excellent vital prognosis and was mostly over-diagnosed [27], so that we excluded it from the malignancy category (Table 1). CCI scores were divided into 3 groups (0, 1, and 2+) for analysis. These CCI scores had been applied in the previous study [28].

#### 2.3.4. Variable of Interest

Four key questionnaire items related to USC were used: for usual place, “Do you have a medical institution that you usually visit when you are ill or when you are trying to get a medical check-up or consultation?”; for type of usual place, “What is the type of medical institution you usually visit?”; for usual physician, “Do you have a medical doctor who you usually see when you are ill or when you are trying to get a medical check-up or consultation?”; and for comprehensiveness of services delivered, “Does the medical doctor solve almost all the common health problems that you have?”. In this study, the authors defined comprehensiveness by whether a usual physician dealt with almost all the common health problems, in spite of hypertension being managed using lifestyle modification and anti-hypertensive medications. Comprehensiveness was rated on a 5-point Likert scale. But, for analysis in this study, ‘very good’ or ‘good’ responses were merged into ‘comprehensive’ and the others into ’non-comprehensive’ for the differentiation of community clinic physicians (CCPs), rather than hospital specialist physicians. Whether having a usual physician (especially, comprehensive community clinic physician) or not was used as a key independent variable in this study.

### 2.4. Statistical Analysis

Types of usual physician of care (not having a usual physician, comprehensive CCPs, non-comprehensive CCPs, and hospital specialists) in adults (18 years or over) with essential hypertension were analyzed descriptively to show distributions by sociodemographic variables, health status variables (SRH status and CCI index score), and a variable of interest using Chi-square tests. Multiple logistic regression analyses were used to identify the association of having a usual physician of care with having ED visits and with hospital admission within a year, and estimate adjusted odds ratios (ORs) of types of usual physicians of care while controlling for sociodemographic and health status variables. Statistical analyses applied by sample-based cross-sectional weights were conducted with SAS version 9.4 software (SAS Institute, Cary, NC, USA). An alpha of 0.05 was used as the cutoff for significance.

## 3. Results

Among adult (≥18 years) panel participants with essential hypertension (*n* = 2627) in this study, 61.9% did not have a usual physician, 21.0% had a comprehensive CCP as a USC, 5.7% had a non-comprehensive CCP as a USC, and 11.4% had a hospital specialist as a USC after applying a weight to each percentage.

### 3.1. Sociodemographic Characteristics of Adults with Essential Hypertension by Types of Physicians as a Usual Source of Care

By bivariate analysis, types of health insurance were significantly associated with types of physicians as a USC. Among those who had a hospital specialist physician as a USC, compared with those who had other types of physician as a USC, Medical Aid or other beneficiaries (13.2%, *p* = 0.029), those who had poor subjective health status (35.2%, *p* = 0.018), those who had 2 or above in CCI score (29.8%, *p* < 0.001), and those who experienced ED visits (13.9%, *p* = 0.033) and hospital admission (26.7%, *p* < 0.001) within a year showed higher percentages. Age, sex, education, household income, and marital status did not have a significant association with types of physician as a USC (Table 2).

### 3.2. Adjusted ORs of Types of Physician as a USC for an Experience of ED Visits Within a Year

After adjusting for sociodemographic factors, those having a comprehensive CCP as a USC (OR: 0.59, 95% CI: 0.39–0.89, *p* = 0.013) were associated with a decrease in ED visits, as compared to those not having a usual physician (model 1). After adjusting for SRH and CCI score as well as sociodemographic factors, those having a comprehensive CCP as a USC (OR: 0.61, 95% CI: 0.40–0.93, *p* = 0.023) remained associated with a decrease in ED visits compared to those not having a usual physician, but those having a non-comprehensive (most likely specialist) CCP or a hospital specialist physician as a USC did not (model 2) (Table 3).

### 3.3. Adjusted ORs of Types of Physician as a USC for an Experience of Hospital Admission within a Year

After adjusting for sociodemographic factors, those having a comprehensive CCP as a USC were associated as well with a decrease (OR: 0.66, 95% CI: 0.48–0.92, *p* = 0.015), while those having a hospital specialist physician as a USC were associated with an increase (OR: 1.80, 95% CI: 1.29–2.51, *p* = 0.001), in hospital admissions compared to those not having a usual physician (model 1). After adjusting for SRH and CCI score as well as sociodemographic factors, those having a comprehensive CCP as a USC (OR: 0.69, 95% CI: 0.49–0.96, *p* = 0.027) remained associated with a decrease, while those having a hospital specialist physician as a USC (OR: 1.52, 95% CI: 1.07–2.14, *p* = 0.018) remained associated with an increase in hospital admissions, compared to those not having a usual physician. Those having a non-comprehensive CCP as a USC did not show a statistical significance, irrespective of adjustments for confounding factors (model 2). The older the age group, the higher the probability of having a hospital admission. Those aged 65 or more years (OR: 2.23, 95% CI 1.28–3.89, *p* = 0.005) were associated with an increase in hospital admissions compared to those aged 18–49 years. Those with 13 years or more in education were associated with a decreased probability of hospital admissions (OR: 0.60, 95% CI: 0.37–0.98, *p* = 0.041) compared to those with 6 years or less in education (model 2) (Table 4).

## 4. Discussion

This study was intended to provide evidence on the value of primary care in the Korean health care system. The authors considered comprehensiveness among 4 core attributes of primary care as a proxy to differentiate primary care from specialist care in the community clinics because first contact and coordination function are not distinctive in Korean primary care, and person-focused care over time (longitudinality) is regarded to be dependent to the other attributes. The authors succeeded in finding 3 new facts related to Korean primary care in this study. In Korean adults (≥18 years) with essential hypertension, after adjusting sociodemographic variables and health status variables (SRH and CCI score), compared with those who do not have a physician as a USC, first, those who had a primary care physician (i.e., comprehensive CCP) as a USC experienced significantly fewer ED visits (OR: 0.61, 95% CI: 0.40–0.93, *p* = 0.023) as well as hospital admissions (OR: 0.69, 95% CI: 0.49–0.96, *p* = 0.027); second, those who had a hospital specialist as a USC demonstrated a significantly higher probability of hospital admissions (OR: 1.52, 95% CI: 1.07–2.14, *p* = 0.018); third, having a non-comprehensive CCP was neither significantly associated with experiences in ED visits nor in hospital admission.

Comprehensiveness, one of the major attributes of primary care, requires that primary care adequately recognize the full range of patients’ health-related needs and provide the resources to meet them. Comprehensiveness is thus judged by the extent to which the available range of services meets needs that are common in all populations and the needs that are common in the population served, as well as the extent to which there is evidence that the services are being applied adequately to meet those needs [29]. However, in Korea, fragmented and episodic care, instead of comprehensive care, is frequent in community care, and first-contact care is provided by all kinds of disease-focused medical specialists and virtually at all levels of medical institutions. Under this poorly defined state of primary care, this study demonstrates that potential primary care (comprehensive community clinic) physicians, rather than non-primary care specialist (non-comprehensive community clinic) physicians, can decrease ED use and hospitalization in Korea, where ED overcrowding and avoidable hospitalization have been a problem to health care quality.

Primary care has an important role in addressing healthcare challenges many countries face, including rising healthcare costs, the increasing prevalence of chronic conditions and multi-morbidity, health inequalities, and potentially avoidable hospitalization [30,31]. Therefore, it has been a trend in many countries to strengthen their primary care systems by several strategies in the last 3 to 5 decades. For example, primary care, represented by general practice in Europe and family medicine in North America, has been a medical specialty that requires post-graduate training (residency) for at least 3 years. Payment in primary care has been changed from traditional fee-for-service scheme to a mixed method including capitation, pay-for-performance payment, or others. Group practice and multidisciplinary team approach have been encouraged in primary care. And, finally, referral system or enrollment in primary care has been established to enhance coordination vertically (between community and hospital care) or horizontally (among various community health professionals) in health care system [32,33,34]. But, Korea, with one of the fastest growing elderly populations among OECD countries, has barely kept up these global trends, except for the introduction of family medicine as a medical specialty. Until now, in contrast, the system has done the opposite: It has encouraged further diagnosis and the utilization of the large hospital sector. Overprovision of treatment has been a major quality of care issue over recent decades in Korea. Compared with other OECD countries, Korea ranks among the highest for potentially preventable admissions relating to COPD, asthma, and uncontrolled diabetes, which underlines the need for targeted actions to ensure that chronic diseases are properly managed within the community setting [35].

This study has some limitations. First, the authors could not help categorizing community care into primary care and specialist care only by comprehensiveness because of the vagueness in the definition of primary care in Korea. We hope that the Korean society can reach a consensus on primary care in the near future. Second, this study could not prove the causal relationship between having a comprehensive PCP as a USC and health care utilization due to a cross-sectional study design. In the future, if more than 2 time periods are available regarding a usual physician in the KHP survey, a longitudinal analysis can be conducted to find the effect on various health outcomes of having a comprehensive PCP. Third, CCI scores in this study might not be calculated exactly because diagnostic codes of the KHP survey consisted of only 3-digits despite it being compatible to the ICD-10 coding system. To compensate for this drawback, SRH was used as one of control variables in multivariate analysis. SRH can comprehensively evaluate a person’s health status and be consistent with objective health status [36]. Fourth, blood pressure level, which might also have a direct impact on the likelihood of ED visits or hospitalization, was not included as a control variable in the multivariate analyses, because it could not be retrieved from the Korea Health Panel data.

## 5. Conclusions

Health care policies that promote having a comprehensive primary care (i.e., community clinic in this study) physician as a USC could decrease unnecessary experiences of ED use and hospitalization by Korean adults with hypertension. This can partly reduce ED overcrowding and avoidable hospitalization in Korea. The international society may learn a lesson from this study’s results and the current situation of Korean primary care.

## Figures and Tables

**Table 1:** Diagnostic codes for calculating Charlson comorbidity index score in adults (≥ 18 years) with hypertension—based on the 2013 Korea Health Panel survey.

Disease	Diagnostic Codes Compatible to the ICD-10 Coding in the KHP Data	CCI Score
Diabetes mellitus	E10–E14	1
Myocardial infarct	I21, I22, I25	1
Congestive heart failure	I50	1
Peripheral vascular disease	I70–I79	1
Cerebrovascular disease	I60–I69	1
Dementia	F03, G30	1
Chronic pulmonary disease	J41, J42, J43, J44, J45, J47, J64	1
Rheumatic or connective tissue disease	M30–M36, M06	1
Gastric or peptic ulcer	K25, K26	1
Mild liver disease	B18, B19, K70–K77	1
Hemiplegia or paraplegia	G80, G81, G82	2
Moderate or severe renal disease	N17–N19	2
Any malignancy, including lymphoma and leukemia, except basal cell cancer of skin	C00–C41, C43, C45–C72, C74, C75, C81–C96	2
Metastatic solid tumor	C76–C80	6
Acquired immune deficiency syndrome	B20-B24	6

ICD: International Classification of Diseases; KHP: Korea Health Panel; CCI: Charlson Comorbidity Index. Two categories are not included in the above CCI score calculation because diagnostic codes for end organ damages of diabetes and for moderate or severe liver diseases are not provided in the KHP data. Thyroid cancer (C73) is excluded from the malignancy category [28].

**Table 2:** Sociodemographic characteristics by types of physicians as a usual source of care in adults (≥18 years) with hypertension—based on 2013 Korea Health Panel survey.

Variables		Total	Not Having a Usual Physician	Comprehensive CCPs	Non-Comprehensive CCPs	Hospital Specialists	*p*
		*n* (%)	*n* (%)	*n* (%)	*n* (%)	*n* (%)	
Age	18–49	224 (12.1)	144 (12.7)	45 (11.4)	15 (12.9)	20 (9.6)	0.370
	50–64	756 (37.1)	457 (36.0)	155 (36.8)	52 (44.8)	92 (39.1)	
	65–	1647 (50.8)	1024 (51.3)	352 (51.9)	83 (42.3)	188 (51.3)	
Sex	Male	1166 (46.8)	728 (47.2)	226 (43.5)	64 (45.0)	148 (52.0)	0.201
	Female	1461 (53.2)	897 (52.8)	326 (56.5)	86 (55.0)	152 (48.0)	
Education (year)	–6	1196 (38.9)	759 (40.3)	249 (38.1)	63 (34.9)	125 (35.0)	0.132
	7–12	1097 (45.0)	670 (43.9)	240 (48.4)	59 (42.5)	128 (46.4)	
	13–	334 (16.1)	196 (15.8)	63 (13.5)	28 (22.6)	47 (18.6)	
Household income (quintile) (missing, 1)	1st (the lowest)	765 (24.2)	460 (23.4)	199 (29.9)	32 (18.9)	74 (21.3)	0.225
	2nd	599 (20.9)	378 (21.1)	116 (19.6)	38 (22.3)	67 (21.7)	
	3rd	507 (19.7)	329 (20.4)	91 (18.1)	32 (21.6)	55 (17.6)	
	4th	410 (17.7)	242 (17.0)	86 (18.2)	25 (17.5)	57 (20.7)	
	5th (the highest)	345 (17.5)	216 (18.1)	60 (14.2)	22 (19.7)	47 (18.7)	
Marital status	Married	1891 (74.0)	1175 (74.0)	389 (72.8)	109 (77.4)	218 (74.7)	0.767
	Others ^1^	736 (26.0)	450 (26.0)	163 (27.2)	41 (22.6)	82 (25.3)	
Health coverage (missing, 1)	Employed	1696 (64.7)	1055 (65.8)	362 (65.9)	94 (58.0)	185 (60.1)	0.029
	Self-employed	690 (26.6)	433 (26.1)	137 (25.0)	46 (36.2)	74 (26.7)	
	Medical Aid or others ^2^	240 (8.7)	136 (8.1)	53 (9.1)	10 (5.8)	41 (13.2)	
Self-rated health (missing, 99)	Good	687 (28.0)	443 (29.5)	150 (29.2)	31 (22.4)	63 (21.8)	0.018
	Moderate	1119 (45.7)	671 (45.2)	252 (46.5)	77 (52.7)	119 (43.0)	
	Poor	722 (26.3)	438 (25.4)	135 (24.3)	39 (24.9)	110 (35.2)	
CCI score	0	1445 (57.5)	937 (59.7)	320 (61.5)	91 (63.2)	97 (35.7)	<0.001
	1	763 (28.1)	449 (27.0)	168 (28.3)	43 (25.7)	103 (34.5)	
	2 or higher	419 (14.4)	239 (13.3)	64 (10.2)	16 (11.1)	100 (29.8)	
ED visit, yearly	Yes	289 (10.6)	188 (11.1)	43 (7.1)	14 (11.5)	44 (13.9)	0.033
	No	2338 (89.4)	1437 (88.9)	509 (92.9)	136 (88.5)	256 (86.1)	
Admission, yearly	Yes	501 (17.2)	307 (16.9)	82 (12.5)	27 (18.7)	85 (26.7)	<0.001
	No	2126 (82.8)	1318 (83.1)	470 (87.5)	123 (81.3)	215 (73.3)	
Total		2627 (100)	1625 (100)	552 (100)	150 (100)	300 (100)	

Chi-square test. CCPs: Community Clinic Physicians; CCI: Charlson Comorbidity Index; ED: Emergency Department. ^1^ Separated, divorced, widowed, and unmarried; ^2^ Out-of-pocket cost reduction for the second tier, health insurance coverage expansion for cancer patients, health insurance benefits for patriots. Cross-sectional weights were applied for percentages and *p* values.

**Table 3:** Association between having a comprehensive community clinic physician (CCP) as a usual source of care and the experience of emergency department (ED) visits within a year in adults (≥18 years) with hypertension—based on the 2013 Korea Health Panel survey.

Variables		Model 1			Model 2		
		OR	95% CI	*p*	OR	95% CI	*p*
Age	18–49	1			1		
	50–64	0.64	0.39–1.04	0.073	0.62	0.37–1.02	0.057
	65–	0.81	0.49–1.34	0.413	0.73	0.44–1.23	0.242
Sex	Male	1			1		
	Female	0.95	0.68–1.32	0.749	0.92	0.66–1.29	0.623
Education (year)	–6	1			1		
	7–12	0.92	0.64–1.32	0.642	0.99	0.68–1.43	0.942
	13–	0.92	0.54–1.59	0.771	1.07	0.62–1.85	0.810
Household income (quintile) (missing, 1)	1st (the lowest)	1			1		
	2nd	1.13	0.74–1.72	0.580	1.12	0.73–1.72	0.600
	3rd	0.85	0.53–1.39	0.523	0.92	0.56–1.50	0.723
	4th	1.27	0.79–2.06	0.326	1.36	0.83–2.21	0.222
	5th (the highest)	0.73	0.41–1.28	0.269	0.75	0.42–1.33	0.328
Marital status	Married	1			1		
	Others	1.32	0.93–1.87	0.118	1.31	0.92–1.86	0.132
Health coverage (missing, 1)	Employed	1			1		
	Self-employed	0.98	0.69–1.37	0.890	0.97	0.69–1.37	0.856
	Medical Aid or others ^1^	1.70	1.02–2.85	0.044	1.36	0.80–2.31	0.261
Self-rated health (missing, 99)	Poor				1		
	Moderate				0.52	0.37–0.73	< 0.001
	Good				0.40	0.27–0.61	< 0.001
Charlson Comorbidity Index score	0				1		
	1				0.85	0.59–1.21	0.362
	2 or higher				1.48	1.00–2.20	0.052
Types of physicians as a USC	Not having a usual physician	1			1		
	CCPs, comprehensive	0.59	0.39–0.89	0.013	0.61	0.40–0.93	0.023
	CCPs, non-comprehensive	1.09	0.61–1.95	0.780	1.08	0.60–1.95	0.803
	Hospital specialists	1.26	0.83–1.91	0.275	1.11	0.73–1.71	0.623
Hosmer and Lemeshow goodness-of-fit test		*p* = 0.834			*p* = 0.183		
Concordance index		*C* = 0.606			*C* = 0.650		

Multiple logistic regression analysis. ED: Emergency Department; CCPs: Community Clinic Physicians; OR: Odds Ratio; CI: Confidence Interval; USC: Usual Source of Care. ^1^ Out-of-pocket cost reduction for the second tier, health insurance coverage expansion for cancer patients, health insurance benefits for patriots. Cross-sectional weights were applied.

**Table 4:** Association between having a comprehensive CCP as a usual source of care and the experience of hospitalization within a year in adults (≥18 years) with hypertension—based on the 2013 Korea Health Panel survey.

Variables		Model 1		Model 2			
		OR	95% CI	*p*	OR	95% CI	*p*
Age	18–49	1			1		
	50–64	1.73	1.01–2.99	0.048	1.64	0.95–2.83	0.078
	65–	2.54	1.47–4.40	0.001	2.23	1.28–3.89	0.005
Sex	Male	1			1		
	Female	0.86	0.66–1.13	0.277	0.84	0.64–1.11	0.227
Education (year)	–6	1			1		
	7–12	0.88	0.66–1.18	0.383	0.93	0.70–1.26	0.650
	13–	0.55	0.34–0.89	0.015	0.60	0.37–0.98	0.041
Household income (quintile) (missing, 1)	1st (the lowest)	1			1		
	2nd	0.79	0.56–1.12	0.187	0.78	0.55–1.10	0.153
	3rd	0.76	0.52–1.11	0.157	0.79	0.54–1.16	0.232
	4th	0.82	0.55–1.23	0.335	0.85	0.57–1.28	0.441
	5th (the highest)	0.71	0.46–1.11	0.138	0.75	0.48–1.17	0.203
Marital status	Married	1			1		
	Others	0.98	0.73–1.32	0.888	0.98	0.73–1.33	0.914
Health coverage (missing, 1)	Employed	1			1		
	Self-employed	0.84	0.63–1.11	0.221	0.81	0.60–1.08	0.143
	Medical Aid or others ^1^	1.41	0.91–2.18	0.130	1.11	0.71–1.75	0.640
Self-rated health (missing, 99)	Poor				1		
	Moderate				0.64	0.48–0.84	0.001
	Good				0.49	0.35–0.68	< 0.001
Charlson Comorbidity Index score	0				1		
	1				1.41	1.07–1.87	0.015
	2 or higher				1.81	1.30–2.53	< 0.001
Types of physicians as a USC	Not having a usual physician	1			1		
	CCPs, comprehensive	0.66	0.48–0.92	0.015	0.69	0.49–0.96	0.027
	CCPs, non-comprehensive	1.26	0.78–2.05	0.342	1.28	0.79–2.09	0.316
	Hospital specialists	1.80	1.29–2.51	0.001	1.52	1.07–2.14	0.018
Hosmer and Lemeshow goodness-of-fit test		*p* = 0.076		*p* = 0.220			
Concordance index		*C* = 0.622		*C* = 0.663			

Multiple logistic regression analysis. OR: Odds Ratio; CI: Confidence Interval; USC: Usual Source of Care; CCPs: Community Clinic Physicians. ^1^ Out-of-pocket cost reduction for the second tier, health insurance coverage expansion for cancer patients, health insurance benefits for patriots. Cross-sectional weights were applied.
